# Does the Fragrance of Essential Oils Alleviate the Fatigue Induced by Exercise? A Biochemical Indicator Test in Rats

**DOI:** 10.1155/2017/5027372

**Published:** 2017-10-31

**Authors:** Zhiyue Li, Fengzhi Wu, Haozhen Shao, Yu Zhang, Angran Fan, Feng Li

**Affiliations:** ^1^School of Traditional Chinese Medicine, Beijing University of Chinese Medicine, Beijing 100029, China; ^2^School of Life Sciences, Beijing University of Chinese Medicine, Beijing 100029, China

## Abstract

**Objective:**

To study the effect of the essential oils of* Citrus sinensis* L.,* Mentha piperita* L.,* Syzygium aromaticum* L., and* Rosmarinus officinalis* L. on physical exhaustion in rats.

**Methods:**

Forty-eight male Wistar rats were randomly divided into a control group, a fatigue group, an essential oil mixture (EOM) group, and a peppermint essential oil (PEO) group. Loaded swimming to exhaustion was used as the rat fatigue model. Two groups were nebulized with EOM and PEO after swimming, and the others were nebulized with distilled water. After continuous inhalation for 3 days, the swimming time, blood glucose, blood lactic acid (BLA), blood urea nitrogen (BUN), superoxide dismutase (SOD), glutathione peroxidase (GSH-PX), and malondialdehyde (MDA) in blood were determined.

**Results:**

While an increased time to exhaustion and SOD activity were apparent in both the EOM and PEO groups, the BLA and MDA were lower in both groups, in comparison with the fatigue group, and the changes in the EOM group were more dramatic. Additionally, the EOM group also showed marked changes of the rise of blood glucose and the decrease of BUN and GSH-PX.

**Conclusion:**

The results suggested that the inhalation of an essential oil mixture could powerfully relieve exercise-induced fatigue.

## 1. Introduction

Physical fatigue will occur sooner or later depending on the type of exercise. Physical fatigue is commonly defined as the inability to sustain or maintain voluntary activity, and it is considered to be associated with a physiological decline [1]. Several theories have been proposed to explain physical fatigue, including the “exhaustion theory,” the “radical theory,” and the “clogging theory.” The “exhaustion theory” proposed that depletion of energy stocks within the body, such as glucose or glycogen, could lead to fatigue. However, the “clogging theory,” that is, the excessive accumulation of blood lactic acid (BLA) and blood urea nitrogen (BUN), would cause metabolic disorders that would eventually result in the fatigue. The “radical theory” hypothesizes that intensive exercise could produce an imbalance between the body's oxidation system and its antioxidation system. Muscle cells have defense mechanisms that scavenge reactive oxygen species (ROS) such as SOD, GSH-Px, and CAT and protect cells against exercise-induced oxidative injury [1–4]. Recovery from exercise-induced fatigue requires repairing the damage that has occurred in the body and eliminating the metabolic products that accumulated during exercise [3].

Essential oils are complex mixtures of strongly odoriferous volatile compounds that are synthesized in various plant organs and have diverse ecological functions [5]. Aromatic essential oils are frequently considered as potential new treatments [6] for various disorders, including fatigue. For instance, Jaradat et al. [7] showed that the inhalation of the* Citrus sinensis* flower essential oil could improve athletic performance.* Citrus sinensis* (L.), also known as orange or sweet orange, is a small tree in the family Rutaceae. The peel oil has antibacterial, antioxidant, or anxiolytic properties [8–10], and the key compound it contains is d-limonene (73.9–97%), along with various amounts of linalool, geraniol, and nerol [11]. A previous study showed that treatment with orange peel essential oil decreased oxidative injury by decreasing the serum malondialdehyde (MDA) level and increasing the activities of antioxidant enzymes in subjects [12], and these effects may help prevent physical fatigue.

Raudenbush et al. [13] showed that peppermint aroma was effective on perceived physical workload, temporal workload, effort, and anxiety. Peppermint (*Mentha piperita*) is a popular herb that can be used in numerous forms (i.e., oil, leaf, leaf extract, and leaf water). The leaf oil has the most uses; it has a variety of therapeutic properties and is used in aromatherapy, bath preparations, mouthwashes, toothpastes, and topical preparations [14]. Menthol (29%) and menthone (20–30%) are the major components of the peppermint leaf essential oil [15]. Inhalation of peppermint essential oil improved the lung capacity and inhalation ability in healthy participants; thus Meamarbashi and Rajabi speculated this effect supply more oxygen to the brain, which could be effective in continuing physical performance [15].

Rosemary has been used in traditional medicine as a stimulant, and it has been considered as one of the most effective herbs for treating physical and mental fatigue [5]. Rosemary (*Rosmarinus officinalis* L., Lamiaceae) is a woody perennial herb, and the highest quality essential oil of* R. officinalis* L. is obtained from the leaves. Rosemary essential oil consists mostly of monoterpenes such as 1,8-cineole (43.77%), camphor (12.53%), and *α*-pinene (11.51%) [16, 17]. Most pharmacological effects of rosemary are a consequence of the high antioxidant activity of its main chemical constituents, which has been mainly attributed to its major diterpenes, carnosol, and carnosic acid, as well as to the essential oil components [18].

*Syzygium aromaticum* (L.), or clove, is a dried unopened flower bud of* Syzygium aromaticum* (L.) Merr. & Perry (Family Myrtaceae) [19]. The essential oil extracted from the dried flower buds of cloves is used as a topical application to relieve pain and to promote healing and is also used in the medical, fragrance, and flavoring industries [20]. The major component of clove essential oil is considered to be eugenol with lesser amounts of *β*-caryophyllene and eugenyl acetate. Eugenol is the primary constituent that has high antiradical activity [21]. In addition to antioxidant activity, clove essential oil increases the content of antibody-forming lymphocyte cells in the spleen [22].

That is, clove essential oil may powerfully protect the cellular structure from being attacked and damaged after exercising to exhaustion.

Exercise-induced fatigue is characterized by two signs: deterioration of performance is the primary sign, and tiredness/exhaustion is the secondary sign. A mixture of four essential oils may be used to treat exercise-induced fatigue: two stimulant essential oils, one relaxing essential oil and a balancing essential oil. Peppermint essential oil can increase alertness and mental clarity and decrease the perceived physical workload [23]. Rosemary essential oil inspires energy and stimulates vitality [5]. Orange essential oil is known for its calming and soothing properties that relax the muscles [12]. As a balancing essential oil, clove essential oil significantly increases the free radical scavenging capacity of the other oils, thus preventing oxidative injury during fatigue. In addition, peppermint and orange essential oils evaporate more quickly than rosemary essential oil, and clove oil volatilizes the slowest, so appropriate proportions of each oil can create a lasting balance.

Previous studies have shown that peppermint essential oil, which is most widely used to prevent fatigue, significantly improves exercise performance [24, 25]. Therefore, peppermint essential oil was used as a positive control in the present study, and its effects were compared to the therapeutic effects of the experimental essential oil mixture. The aim of this study was to assess the antiphysical fatigue effects of supplementation with the essential oil mixture by inhalation on the time to exhaustion, blood glucose, BLA, and other biochemical parameters.

## 2. Materials and Methods

### 2.1. Chemicals and Reagents

A glucose assay kit was purchased from the Shanghai Rongsheng Biotech Co., Ltd. (Shanghai, China). A blood urea nitrogen (BUN) kit, blood lactic acid (BLA) kit, superoxide dismutase (SOD) kit, glutathione peroxidase (GSH-PX) kit, and malondialdehyde (MDA) kit were from Nanjing Jiancheng Biotechnology Institute (Nanjing, China). All other chemicals used were of analytical grade.

### 2.2. Preparation of Essential Oils

Peppermint essential oil, orange sweet essential oil, and rosemary essential oil were purchased from the Beijing Maosi Commercial and Trading Co., Ltd. (Beijing, China). The clove essential oil came from Aroma Zone Distributors, France. The countries of origin were France and Spain (peppermint); the US and Australia (orange); Spain and France (rosemary); and Madagascar (clove). The material safety data sheets were supplied by http://www.afuvip.com or https://www.aroma-zone.com.

The essential oil mixture was made up of* Citrus sinensis*/orange peel oil,* Mentha piperita*/peppermint leaf oil,* Syzygium aromaticum*/clove bud oil, and* Rosmarinus officinalis*/rosemary flower and leaf oil in equal amounts. The positive control inhalers contained peppermint leaf essential oil.

### 2.3. GC-MS Analysis

The essential oils were analyzed by gas chromatography-mass spectrometry (GC-MS) using a Shimadzu GCMS-QP 2010 gas chromatograph-mass spectrometer (Kyoto, Japan). The instrument conditions were as follows: GC conditions: analytical column: HP-5MS 30 m × 0.25 mm, 0.25 *μ*m 5% phenyl methyl silicone; injection temperature: 250°C; injection mode: split ratio set to 20 : 1; oven program: 2 min at 50°C, 8°C/min to 240°C; column flow: 1 ml/min constant flow; carrier gas: helium. MS conditions were as follows: ionization mode: electron impact at 70 eV; ion source temperature: 230°C; transfer line temperature: 280°C; acquisition mode: scan (30–550 amu).

### 2.4. Identification of Components

The percentage composition of the essential oils was computed from the GC peak areas. Most constituents were tentatively identified by comparison of their GC Kovats retention indices (RI), determined with reference to a homologous series of C5–C28 n-alkanes and with those of authentic standards. Further identification was made by matching with compounds in a NIST Mass Spectral Library.

### 2.5. Animals

#### 2.5.1. Animals and Care

A total of 48 adult male Wistar rats (200 ± 20 g) were purchased from the Beijing Vital River Laboratory Animal Technology Co., Ltd. (Certificate: SCXK (Beijing) 2012-0001). All rats were housed in conventional cages in a temperature (22°C–24°C) and light-controlled (12-hour light- dark cycle) room for 7 days [26] with standard rat chow and water ad libitum [27]. The experimental protocol was approved by the local Animal Care Committee at the Beijing University of Chinese Medicine. All the experimental procedures were carried out in accordance with the international guidelines for the care and use of laboratory animals.

#### 2.5.2. Grouping of Animal

The animals were randomly divided into 4 groups, with 12 rats in each group. The groups were the control group, the fatigue group, the essential oil mixture (EOM) group, and the peppermint essential oil (PEO) group. The EOM and PEO groups were, respectively, nebulized with a mixture of essential oils and peppermint oil (0.4 ml/kg of body mass of each kind of oil) mixed with 2 ml of distilled water, according to the method validated by Chen et al. [28]. The control group and fatigue group were nebulized with an equal volume of distilled water. All groups were nebulized 10 minutes after the end of swimming to exhaustion.

#### 2.5.3. Rat Fatigue Model

Loaded swimming to exhaustion was selected as the fatigue model and evaluation index. Xiaoming et al. [29] noted that swimming has more advantages than other exercises such as the treadmill because training is not required since rodents have a natural swimming ability, and they would try to struggle to avoid drowning even when fatigue is extreme, assuring a high level of performance.

After 7 days acclimation, the weight loaded swimming to exhaustion was carried out as described by Chi et al. [3] with slight modifications. All of the rats were removed from the fatigue group, EOM group, and PEO group for the swimming to exhaustion in a pool (length 65 cm, width 50 cm, depth 50 cm, water depth 30 cm, and temperature 25 ± 1°C). A lead block (10% of body weight) was attached to the tail root of the swimming rat [30]. The rats were determined to be exhausted when they failed to rise to the surface to breathe after 7 s [30]. The rats in the control group were normally raised without any treatment until the end of the experiment. Swimming to exhaustion started at 5 p.m. every day for three days. All rats were fasted 12 hours before bleeding.

### 2.6. Swimming to Exhaustion Test

All the rats were removed from each experimental group and made to swim while carrying a load. The swimming equipment and methods are consistent with the previously mentioned protocol.

The standard for exhaustion was submersion in the water for 7 s without the surfacing of the head. When a rat was placed on a plane, it was not able to complete the righting reflex. The swimming time was immediately recorded.

### 2.7. Assay of Biochemical Parameters

After swimming, a blood sample was collected from the rats in a common tube by abdominal aortic access for the determination of blood glucose, BUN, BLA, SOD, GSH-PX, and MDA. The determination and method of operation were performed according to the recommended procedures provided with the kits.

### 2.8. Statistical Analysis

The SPSS 17.0 software was utilized for the statistical analyses. The results were expressed as the mean ± standard deviation (SD). Differences between the treatment means were analyzed with Tukey's test for multiple comparisons. *P* < 0.05 indicated a significant difference.

## 3. Results

### 3.1. Chemical Composition of the Essential Oils

The results of chemical analysis of the essential oils were as follows. The relative contents of the primary components in peppermint leaf essential oil were pulegone (1.17%), menthofuran (2.11%), menthone (25.42%), menthol (38.81%), menthyl acetate (4.38%), and 1,8-cineole (5.57%). The primary components in orange peel essential oil were limonene (94.64%), myrcene (1.91%), *α*-pinene (0.47%), linalool (0.30%), and decanal (0.17%). In rosemary flower and leaf essential oil, the primary components were 1,8-cineole (47.42%), *α*-pinene (13.62%), camphor (11.13%), *β*-pinene (4.39%), and borneol (2.55%). The primary components in clove bud essential oil were eugenol (82.50%), *β*-caryophyllene (3.89%), and eugenyl acetate (12.07%).

### 3.2. The Effect of the EOM on Time to Swim to Exhaustion of Rat

**Figure 1:**
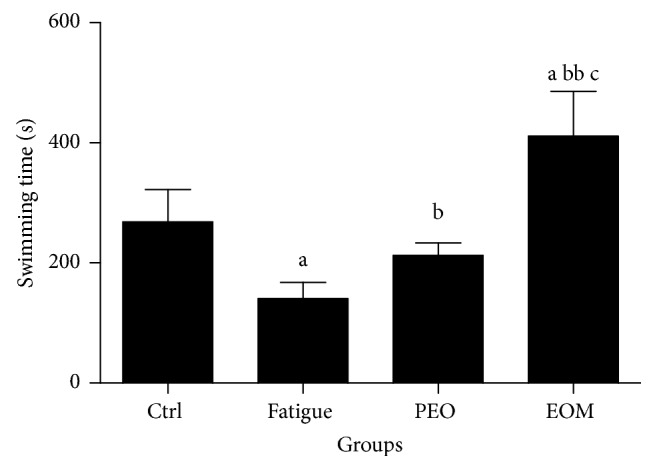
*The effects of the EOM on the time to swim to exhaustion of rats*.* Notes*. ^a^*P* < 0.05 versus the control group; ^b^*P* < 0.05, ^bb^*P* < 0.01 versus the fatigue group; ^c^*P* < 0.05 versus the PEO group; EOM: essential oil mixture; PEO: peppermint essential oil.

Figure 1 shows that the time to swim to exhaustion for the fatigue group was shorter than that for the control group (*P* < 0.05). However, the time to swim to exhaustion of the EOM group was significantly longer than for the other three groups (*P* < 0.05 or *P* < 0.01).

### 3.3. The Effect of the EOM on the Biochemical Parameters of Rats

**Figure 2:**
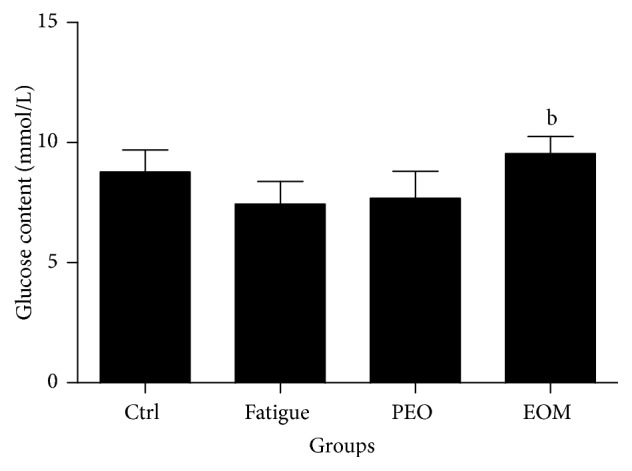
*The effects of the EOM on the blood glucose of rats after swimming*.* Notes*. ^b^*P* < 0.05 versus fatigue group; EOM: essential oil mixture; PEO: peppermint essential oil.

Figure 2 shows that no significant difference in blood glucose was apparent between the control group, the fatigue group, and the PEO group (*P* > 0.05), but the blood glucose of rats in the EOM group was significantly higher than that of the fatigue group after swimming (*P* < 0.05).

**Figure 3:**
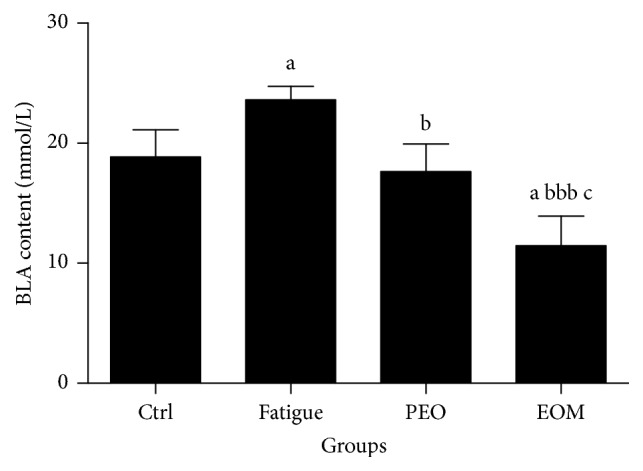
*The effects of the EOM on the BLA of rats after swimming*.* Notes*. ^a^*P* < 0.05 versus the control group; ^b^*P* < 0.05, ^bbb^*P* < 0.001 versus the fatigue group; ^c^*P* < 0.05 versus the PEO group; EOM: essential oil mixture; PEO: peppermint essential oil; BLA: blood lactic acid.

**Figure 4:**
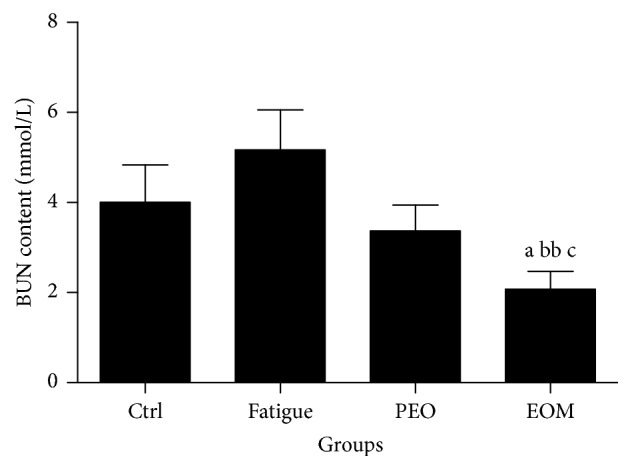
*The effects of the EOM on the BUN of rats after swimming*.* Notes*. ^a^*P* < 0.05 versus the control group; ^bb^*P* < 0.01 versus the fatigue group; ^c^*P* < 0.05 versus the PEO group; EOM: essential oil mixture; PEO: peppermint essential oil; BUN: blood urea nitrogen.

Figures 3 and 4 show that the BLA level of the rats in the fatigue group was significantly higher than that of the control group after swimming (*P* < 0.05). However, inhalation of the EOM significantly decreased the concentrations of BUN and BLA in comparison with the fatigue group (*P* < 0.01 or *P* < 0.001). The BUN and BLA concentrations were even lower than the PEO group and the control group (*P* < 0.05).

**Figure 5:**
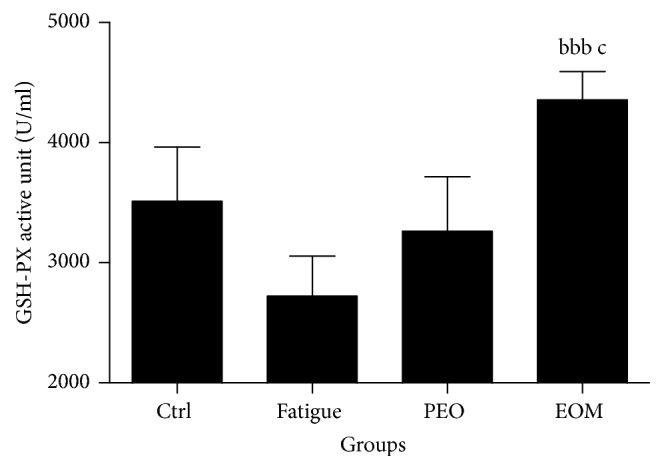
*The effects of the EOM on GSH-PX of rats after swimming*.* Notes*. ^bbb^*P* < 0.001 versus the fatigue group; ^c^*P* < 0.05 versus the PEO group; EOM: essential oil mixture; PEO: peppermint essential oil; GSH-PX: glutathione peroxidase.

**Figure 6:**
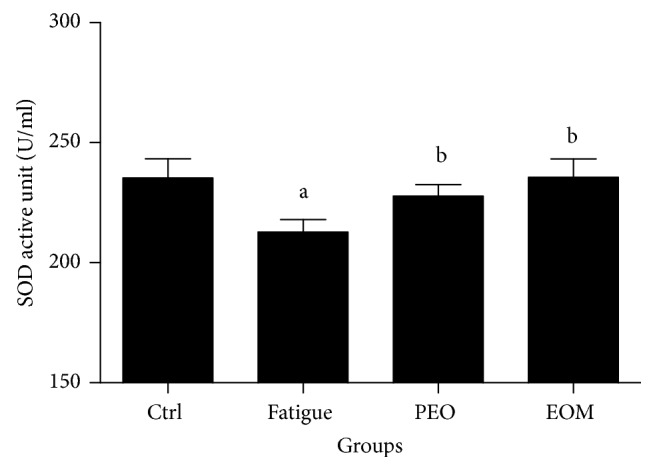
*The effects of the EOM on SOD in rats after swimming*.* Notes*. ^a^*P* < 0.05 versus the control group; ^b^*P* < 0.05 versus the fatigue group; EOM: essential oil mixture; PEO: peppermint essential oil; SOD: superoxide dismutase.

Figures 5 and 6 show that the activity of GSH-PX and SOD (*P* < 0.05) in the fatigue group was lower than in the control group, but the activity of SOD and GSH-PX in the EOM group was higher than in the fatigue group and the PEO group (*P* < 0.05 or *P* < 0.001).

**Figure 7:**
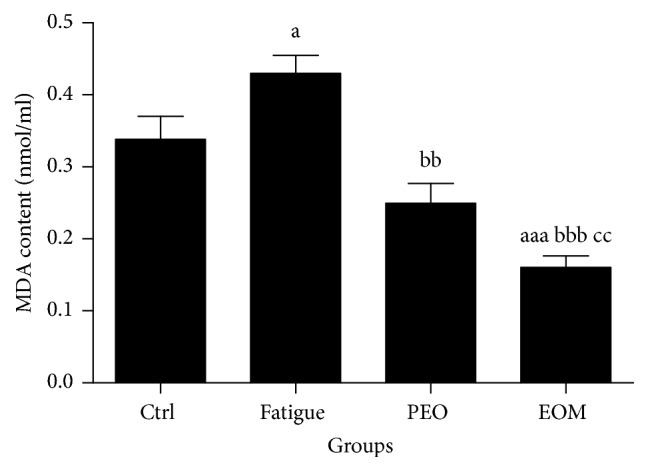
*The effects of the EOM on MDA in rats after swimming*.* Notes*. ^a^*P* < 0.05, ^aaa^*P* < 0.001 versus the control group; ^bb^*P* < 0.01, ^bbb^*P* < 0.001 versus the fatigue group; ^cc^*P* < 0.01 versus the PEO group; EOM: essential oil mixture; PEO: peppermint essential oil; MDA: malondialdehyde.

Figure 7 shows that the MDA in rats in the fatigue group was significantly higher than in the control group after swimming (*P* < 0.05). However, EOM treatment significantly decreased the MDA in comparison with the other groups (*P* < 0.01 or *P* < 0.001).

## 4. Discussion

Since weight loaded swimming is an ideal experimental model to evaluate the antifatigue capacity [29], the length of time to swim to exhaustion indicates the degree of fatigue [31]. Figure 1 shows that the time to exhaustion for rats in the fatigue group was significantly shorter than for those in the control group, indicating that the model was valid. EOM treatment remarkably prolonged the time to exhaustion in rats compared with the other three groups, suggesting that EOM has antifatigue effects superior to those of PEO.

Under normal conditions, glycogen in the liver and muscle is broken down into glucose, and glucose supplies energy to exercising muscles via blood circulation. Energy use leads to the reduction of serum glucose, and the level of blood glucose is a key factor for evaluating the time to exhaustion due to exercise [32]. Figure 2 shows that the EOM reduced glucose consumption, which indicates it could decrease the depletion of the energy source.

BLA is glycolytic product of carbohydrate under anaerobic conditions, and glycolysis is the principal pathway that provides an energy source for intensive exercise for a short time [29]. The increase of the BLA concentration and the consequent lactic acidosis observed in skeletal muscles during exercise were the major cause of muscle fatigue [3]. The results of the present study showed that EOM treatment reduced the BLA and improved glucose metabolism.

BUN is a metabolic product of proteins and amino acids and is a blood biochemical parameter related to fatigue. In general, the body will breakdown proteins only when it cannot get enough energy from glucose and fat. Therefore, BUN is often used to assess the physical load on the body. It is often used as a sensitive experimental index to evaluate exercise tolerance. The less adapted an animal is to exercise, the more the BUN level increases [3]. The results of the present study showed that EOM treatment reduced the serum BUN level and indicated that the EOM had a positive effect on enhancing endurance.

Oxidative stress should be responsible for muscle fatigue [33]. The antioxidant system in the body is weak in exercise-fatigue conditions [34], and the SOD and GSH-Px activities are generally considered indicators of the capacity of the antioxidant defensive system [3]. Malondialdehyde (MDA) is an end-product of the radical-initiated oxidative decomposition of cell membrane lipids, so it is frequently used as a biomarker of oxidative stress [35]. Figures 6 and 7 show that, compared with the control group, the SOD activity decreased but the serum MDA level increased in the fatigue group. Additionally, the data showed that EOM treatment reduced the serum MDA and increased the activities of SOD and GSH-Px after swimming to exhaustion. The present study showed that the muscle could have been assaulted by ROS after exercising to exhaustion. It additionally suggested that the antifatigue effect of the EOM probably occurred via protection of the cell membrane by preventing lipid oxidation due to the modification of the GSH-Px and SOD activities.

## 5. Conclusion

In conclusion, our results suggested that the inhalation of the essential oils of* Citrus sinensis* L.,* Mentha piperita* L.,* Syzygium aromaticum* L., and* Rosmarinus officinalis* L. could powerfully relieve exercise-induced fatigue and the antiphysical fatigue effect of the inhaled EOM was better than that of peppermint essential oil. The putative antifatigue mechanism is the inhibition of the production and accumulation of metabolites, indicated by the reduction of the BUN and BLA in the present study, the decreased depletion of the energy source, indicated by the reduction of glucose depletion, and the protection of the cell structure from attack and damage after exercising to exhaustion by the high antioxidant activity, as indicated by the enhanced activity of the antioxidant enzymes. The results provide an experimental basis for developing a new sort of natural antifatigue spray. They also indicate that the EOM could be used to achieve antiphysical fatigue effects by sniffing. However, further study is needed to clarify the antifatigue mechanism at the cellular and molecular level.

## Abbreviations

EOM: The essential oil mixture
PEO: The peppermint essential oil
BUN: Blood urea nitrogen
BLA: Blood lactic acid
ROS: Reactive oxygen species
GSH-PX: Glutathione peroxidase
SOD: Superoxide dismutase
CAT: Catalase
MDA: Malondialdehyde.
